# Prognostic factors of lenvatinib plus pembrolizumab therapy for advanced or recurrent endometrial cancer: analysis of a multicenter cohort study in Japan

**DOI:** 10.1007/s10147-025-02842-x

**Published:** 2025-09-06

**Authors:** Yoshikazu Nagase, Satoshi Nakagawa, Mariya Kobayashi, Hiroki Kurahashi, Hiromi Ogimoto, Ayaka Tanaka, Tomoko Tsujie, Mayu Shiomi, Akiko Otake, Kanji Masuhara, Kenichi Yoshikawa, Fusanori Yotsumoto, Tomoko Kurita, Kiyoshi Yoshino, Emi Yoshioka, Tomomi Egawa-Takata, Wataru Kudaka, Masayuki Sekine, Hikari Unno, Masahiko Takemura, Saki Aso, Kentaro Kai, Eiji Kobayashi, Takeshi Yokoi, Masashi Akada, Reisa Kakubari, Tsuyoshi Hisa, Shinya Matsuzaki, Yutaka Ueda

**Affiliations:** 1 Department of Obstetrics and Gynecology, Kaizuka City Hospital, Osaka, Japan; 2 Department of Obstetrics and Gynecology, The University of Osaka Graduate School of Medicine, 2-2 Yamadaoka, Suita, Osaka 565-0871 Japan; 3 Department of Obstetrics and Gynecology, Itami City Hospital, Hyogo, Japan; 4 Department of Obstetrics and Gynecology, Nippon Life Hospital, Osaka, Japan; 5 Department of Obstetrics and Gynecology, Toyonaka Municipal Hospital, Osaka, Japan; 6 Department of Obstetrics and Gynecology, Osaka Keisatsu Hospital, Osaka, Japan; 7 Department of Obstetrics and Gynecology, Minoh City Hospital, Osaka, Japan; 8 Department of Obstetrics and Gynecology, Hyogo Prefectural Nishinomiya Hospital, Hyogo, Japan; 9 Department of Obstetrics and Gynecology, Faculty of Medicine, Fukuoka University, Fukuoka, Japan; 10 Department of Obstetrics and Gynecology, School of Medicine, University of Occupational and Environmental Health, Fukuoka, Japan; 11 Department of Obstetrics and Gynecology, Kansai Rosai Hospital, Hyogo, Japan; 12 Department of Obstetrics and Gynecology, Graduate School of Medicine, University of the Ryukyus, Okinawa, Japan; 13 Department of Obstetrics and Gynecology, Osaka General Medical Center, Osaka, Japan; 14 Department of Obstetrics and Gynecology, Faculty of Medicine, Oita University, Oita, Japan; 15 Department of Gynecology, Osaka International Cancer Institute, Osaka, Japan

**Keywords:** Endometrial cancer, Lenvatinib, Pembrolizumab, Platinum-free interval, Prognostic factors

## Abstract

**Background:**

Lenvatinib plus pembrolizumab (LP) therapy has emerged as an effective treatment for patients with advanced or recurrent endometrial cancer. However, limited data are available regarding its outcomes in real-world settings. This study aimed to identify prognostic factors associated with the efficacy of LP therapy.

**Methods:**

This multicenter observational study was conducted across 15 institutions in Japan and examined patients with endometrial cancer, including uterine carcinosarcoma, who experienced disease progression after receiving at least one platinum-based chemotherapy, including adjuvant treatment, and subsequently received LP therapy. The prognostic factors for progression-free survival were assessed using a multivariate Cox proportional hazards model.

**Results:**

A total of 105 patients met the inclusion criteria. Improved progression-free survival was independently associated with performance status of 0 (adjusted hazard ratio [aHR] 0.42, 95% confidence interval [CI] 0.23–0.75), platinum-free interval (PFI) of ≥ 6 months (aHR 0.46, 95% CI 0.28–0.78), histology of grade 1–2 endometrioid carcinoma (aHR 0.52, 95% CI 0.30–0.91), and relative dose intensity during the initial 8 weeks (8w-RDI) of lenvatinib of ≥ 50% (aHR 0.53, 95% CI 0.31–0.91). Patients with PFI of ≥ 6 months also demonstrated improved overall survival (HR 0.44, 95% CI 0.25–0.76) and objective response rate (44.0% versus 20.0%, *P* = 0.011) compared with those with PFI of < 6 months. Additionally, 8w-RDI of lenvatinib ≥ 50% was associated with improved overall survival (HR 0.53, 95% CI 0.30–0.92) compared to those with < 50%.

**Conclusions:**

This study identified several novel prognostic factors for LP therapy. Among them, PFI may inform treatment selection for recurrent endometrial cancer following chemotherapy.

**Clinical trial registration:**

University Hospital Medical Information Network Clinical Trials Registry (UMIN-CTR) 000049997.

**Supplementary Information:**

The online version contains supplementary material available at 10.1007/s10147-025-02842-x.

## Introduction

Endometrial cancer (EC) is increasingly prevalent worldwide, with an incidence rate of 8.4/100,000 [1–3]. Although most patients are diagnosed at an early stage [4], the prognosis of those with advanced or recurrent EC remains poor. For patients with stage IVB disease, platinum-based chemotherapy serves as the mainstay of treatment; however, the 5-year overall survival (OS) rate remains low (20–30%) [3, 5]. In cases of recurrence following first-line platinum-based chemotherapy, available second-line treatment options remain limited, and platinum-based regimens are frequently re-administered. However, several retrospective studies conducted in Japan have demonstrated that a short platinum-free interval (PFI) was associated with reduced effectiveness of second-line chemotherapy in patients with recurrent EC [6, 7].

The randomized phase III Study 309/KEYNOTE-775 trial demonstrated that lenvatinib plus pembrolizumab (LP) significantly improved progression-free survival (PFS) (hazard ratio [HR] 0.56, 95% confidence interval [CI] 0.48–0.66) and OS (HR 0.65, 95% CI 0.55–0.77) compared with conventional chemotherapy in patients with advanced or recurrent EC [8, 9]. This trial included patients with advanced or recurrent EC who had received at least one line of platinum-based chemotherapy, excluding those with uterine carcinosarcoma (UCS). Based on these results, LP has been established as an effective treatment option for this patient population. However, this phase III trial did not investigate factors influencing the therapeutic efficacy of LP. Thus, clinical questions remain regarding the appropriate selection of patients for LP therapy as opposed to platinum rechallenge or alternative treatment regimens. This study aimed to analyze data from Japanese patients with advanced or recurrent EC treated with LP in a real-world setting and identify the prognostic factors for LP therapy.

## Patients and methods

### Study design and data source

This multicenter observational study was conducted at 15 institutions in Japan designated as the Gynecologic Oncology Group of Osaka (GOGO). Ethical approval was obtained from the Institutional Review Board of the University of Osaka, the host institution (approval number 22081[T20]), and the respective review boards of all participating GOGO institutions. This study was registered with the University Hospital Medical Information Network Clinical Trials Registry (UMIN-CTR: 000049997) and conducted in accordance with the Declaration of Helsinki. Written informed consent was obtained from all participants. The Strengthening the Reporting of Observational Studies in Epidemiology guidelines were consulted to outline this observational cohort study [10].

### Eligibility criteria

Consecutive patients with advanced or recurrent EC, including UCS, who received LP therapy between January 2022 and February 2024 were eligible for this study. All patients had experienced disease progression following at least one platinum-containing chemotherapy regimen, including neoadjuvant or adjuvant chemotherapy, and had an Eastern Cooperative Oncology Group performance status (ECOG-PS) of 0–1. Patients with sarcoma (e.g., uterine leiomyosarcoma or endometrial stromal sarcoma), active concurrent cancer, or a history of immune checkpoint inhibitor (ICI) use prior to LP treatment initiation were excluded. Patients for whom lenvatinib intake could not be accurately monitored—due to poor adherence to medication, irregular clinic visits, or insufficient medical records—were also excluded from the study.

### Analysis of outcome measures

The primary outcomes were PFS and the prognostic factors associated with PFS. The secondary outcome was OS, whereas the co-secondary outcomes included tumor response and adverse events (AEs).

To assess these outcomes, we analyzed the following clinical characteristics and findings: (1) age, (2) ECOG-PS, (3) histological type and grade, (4) stage according to the 2008 International Federation of Gynecology and Obstetrics staging system, (5) mismatch repair (MMR) status, (6) number of previous chemotherapy regimens, (7) PFI, (8) the relative dose intensity during the initial 8 weeks of treatment (8w-RDI) of lenvatinib, (9) AEs, (10) best overall response (BOR) during LP therapy, (11) post-LP treatment, (12) date of disease progression, and (13) date of death or last contact.

### Study definitions

PFS was defined as the interval from the initiation of LP therapy to either disease progression or death. OS was defined as the time from the initiation of LP therapy to death from any cause. Patients who had not experienced a survival event at the time of the last follow-up were censored.

The BOR during LP therapy was assessed according to the Response Evaluation Criteria in Solid Tumors (RECIST) version 1.1 or iRECIST guidelines [11, 12]. Patients without measurable target lesions who did not achieve a complete response (CR) or develop progressive disease (PD) were classified as having non-CR/non-PD. The objective response rate (ORR) was defined as the proportion of patients who achieved CR or partial response (PR). The disease control rate (DCR) was defined as the proportion of patients with CR, PR, stable disease, or non-CR/non-PD. Duration of response (DOR) was defined as the period from the first documentation of CR or PR until disease progression or death.

AEs were graded based on the National Cancer Institute Common Terminology Criteria for Adverse Events, version 5.0 [13]. In this study, the recommended initial doses for LP therapy were 20 mg/day of lenvatinib and 200 mg of pembrolizumab administered every three weeks. The dose of LP was reduced, interrupted, or discontinued at the discretion of the attending physician, based on the occurrence of AEs or the oncological response. In the phase III Study 309/KEYNOTE-775 trial, tumor responses were most apparent during the first 8 weeks [8, 9]; therefore, this study examined the 8w-RDI of lenvatinib. The 8w-RDI of lenvatinib was defined as the ratio of the dose administered in the first 8 weeks to the recommended dose (20 mg/day for 8 weeks).

### Statistical analysis

Survival analyses, including PFS, OS, and DOR, were performed using the Kaplan–Meier method and log-rank test. Continuous variables were analyzed using the Student’s *t* test or the Mann–Whitney *U* test, as appropriate. Categorical variables were evaluated using the chi-square or Fisher’s exact test. HRs and 95% CIs were estimated using the Cox proportional hazards regression model. JMP Pro software version 16.0.0 (SAS Institute, Cary, NC, USA) was used for all analyses. Statistical significance was set at *P* < 0.05.

## Results

### Patient characteristics

The characteristics of the 105 enrolled Japanese patients are summarized in Table 1. The median follow-up period was 11.3 months (range 1.8–33.0). The median age of the patients at the initiation of LP therapy was 64 years (range 30–84). The chemotherapy regimens administered prior to LP therapy are provided in Supplementary Table S1. All patients had previously received chemotherapy consisting of either a taxane-platinum combination or a platinum-anthracycline combination. Among the 57 patients who received one prior line of chemotherapy, 50 had received it as adjuvant treatment. Of the 105 eligible patients, 83 (79.0%) discontinued LP therapy during the observation period, with a median treatment duration of 6.3 months (range 0.1–26.4). The reasons for discontinuation included disease progression in 40 patients, AEs in 38, achievement of CR in two, and patient request in three. Six patients who achieved CR during the observation period but continued LP therapy at the discretion of the treating physician.

**Table 1:** Patient characteristics

Number of cases	105
Age	64 (30–84)
ECOG-PS	
0	85 (81.0)
1	20 (19.0)
Histological type	
Endometrioid carcinoma, grade 1–2	43 (41.0)
Endometrioid carcinoma, grade 3	13 (12.4)
Serous carcinoma	24 (22.9)
Carcinosarcoma	10 (9.5)
Other	15 (14.3)
Mix (endometrioid and serous carcinoma)	4 (3.8)
Clear cell carcinoma	4 (3.8)
Dedifferentiated carcinoma	4 (3.8)
Mucinous carcinoma	1 (1.0)
Mesonephric-like adenocarcinoma	1 (1.0)
Undifferentiated carcinoma	1 (1.0)
FIGO stage (2008) at initial treatment	
I–II	37 (35.2)
III–IV	68 (64.8)
Recurrent lesion site	
Lymph node	54 (51.4)
Intra-abdominal	58 (55.2)
Lung	32 (30.5)
Liver	7 (6.7)
Bone	9 (8.6)
MMR status	
MMR-deficient	4 (3.8)
MMR-proficient	60 (57.1)
Unknown	41 (39.0)
Prior lines of chemotherapy	
1	57 (54.3)
2	29 (27.6)
3 ≤	19 (18.1)
Platinum-free interval	
< 6 months	55 (52.4)
≥ 6 months, < 12 months	29 (27.6)
≥ 12 months	21 (20.0)
Prior neoadjuvant and/or adjuvant chemotherapy	86 (81.9)
Prior radiation therapy	25 (23.8)

Number (percent per column) or median (range) is shown

*ECOG-PS* Eastern Cooperative Oncology Group performance status, *FIGO* the International Federation of Gynecology and Obstetrics, *MMR* mismatch repair

Twenty patients received chemotherapy following LP therapy; among them, 14 were treated with platinum-based combination therapy, four with doxorubicin monotherapy, and one with pembrolizumab retreatment. After LP therapy, three patients underwent radiotherapy, while two received hormonal therapy (Supplementary Table S2).

### Survival outcomes and tumor response

The median PFS and OS for all enrolled patients were 8.8 months (95% CI 6.8–11.5; number of events: 72) and 15.1 months (95% CI 12.8–20.3; number of events: 55), respectively. The ORR for LP therapy was 31.4%, whereas the DCR was 76.2%. Among patients who achieved a CR or PR, the median DOR was 13.8 months (95% CI 6.5–not available [NA]), and the median time to response was 3.1 months (range 1.1–26.0).

Of the 105 patients, 22 had no measurable target lesions, and 3 discontinued LP therapy because of AEs before their initial radiology assessment. Of the 80 patients with measurable lesions and imaging evaluations, the median best percentage change from baseline was − 14.9% (interquartile range [IQR] − 50.8% to + 6.5%), indicating tumor shrinkage in 51 patients (63.8%) (Supplementary Figure S1).

### Effect of histological types and MMR status on PFS

The effect of histological type on PFS is presented in Supplementary Figure S2. Patients with non-grade 1–2 endometrioid carcinoma exhibited shorter PFS compared with those with grade 1–2 endometrioid carcinoma (HR 2.12, 95% CI 1.29–3.48). Specifically, PFS was significantly decreased in patients with grade 3 endometrioid carcinoma (HR 2.86, 95% CI 1.39–5.91) and UCS (HR 3.07, 95% CI 1.35–6.95) compared with those with grade 1–2 endometrioid carcinoma. However, the reduction in PFS observed in patients with serous carcinoma was not statistically significant (HR 1.74, 95% CI 0.92–3.30).

Patients with MMR-deficient tumors tended to exhibit longer PFS compared with those with MMR-proficient or unknown MMR status. However, the difference was not statistically significant (not reached [95% CI 2.9–NA] versus 8.7 months [95% CI 6.8–11.5], *P* = 0.30; Supplementary Figure S3).

### Association of PFI with survival outcomes and tumor response

**Fig. 1:**
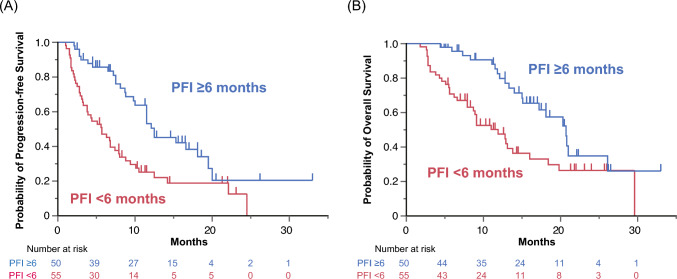
Survival outcomes based on the platinum-free interval in patients treated with lenvatinib plus pembrolizumab. Kaplan–Meier curves for **A** progression-free survival and **B** overall survival are shown. *PFI* platinum-free interval

The median PFS was 12.5 months (95% CI 10.0–18.1) in patients with a PFI of ≥ 6 months (*n* = 50), compared with 5.7 months (95% CI 3.3–7.9) in those with a PFI of < 6 months (*n* = 55) (HR 0.44, 95% CI 0.27–0.70, Fig. 1A). The median OS was 20.7 months (95% CI 15.1–26.1) in patients with a PFI of ≥ 6 months and 11.9 months (95% CI 7.9–16.0) in those with a PFI of < 6 months (HR 0.44, 95% CI 0.25–0.76, Fig. 1B).

**Fig. 2:**
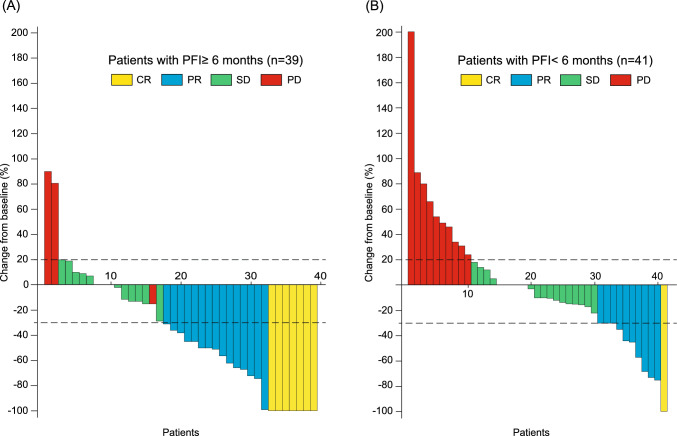
Best percentage change from baseline for target lesions. Waterfall plots demonstrate the best percentage change from baseline for measurable target lesions, based on assessments per RECIST version 1.1 or iRECIST, in patients **A** with a PFI of ≥ 6 months and **B** with a PFI of < 6 months. *PFI* platinum-free interval, *CR* complete response, *PR* partial response, *SD* stable disease, *PD* progressive disease, *RECIST* Response Evaluation Criteria in Solid Tumors

Compared with patients with a PFI of < 6 months, those with a PFI of ≥ 6 months demonstrated a significantly higher ORR (44.0% versus 20.0%, *P* = 0.011) and showed a higher DCR (82.0% versus 70.9%, *P* = 0.25) (Table 2). Among patients who achieved a CR or PR, the median DOR was significantly longer in the PFI ≥ 6 months group compared with the PFI < 6 months group (not reached [95% CI 6.8–NA] versus 4.5 months [95% CI 2.0–NA], *P* < 0.01; Supplementary Figure S4). However, the median time to response was comparable between the two groups (3.05 months [range 1.3–26.0] versus 3.1 months [range 1.1–17.8]; Mann–Whitney *U* test, *P* = 0.97). Among patients with measurable lesions who underwent imaging evaluations (*n* = 80), the median tumor shrinkage rate was significantly greater in the PFI ≥ 6 months group (− 38.0%, IQR − 72.0% to 0%) compared with the PFI < 6 months group (− 10.0%, IQR − 30.0% to + 21.0%; Student’s *t* test, *P* < 0.01). Furthermore, the proportion of patients exhibiting tumor shrinkage tended to be higher in the PFI ≥ 6 months group compared with the PFI < 6 months group (74.4% [29/39 patients] versus 53.7% [22/41 patients], *P* = 0.066) (Fig. 2).

**Table 2:** Relationship between tumor response and platinum-free interval in patients receiving lenvatinib plus pembrolizumab

	Overall	Platinum-free interval		
		≥ 6 months	< 6 months	*P* value
Number of patients	105	50	55	
Best overall response, *n* (%)				
CR	8 (7.6)	7 (14.0)	1 (1.8)	
PR	25 (23.8)	15 (30.0)	10 (18.2)	
SD	33 (31.4)	13 (26.0)	20 (36.4)	
Non-CR/non-PD	14 (13.3)	6 (12.0)	8 (14.5)	
PD	22 (21.0)	7 (14.0)	15 (27.3)	
Not assessed	3 (2.9)	2 (4.0)	1 (1.8)	
Objective response rate, %	31.4	44.0	20.0	0.011
Disease control rate, %	76.2	82.0	70.9	0.25

*CR* complete response, *PR* partial response, *SD* stable disease, *PD* progressive disease

### Relationship between the RDI of lenvatinib and outcomes

Among patients included in this study, the median 8w-RDI of lenvatinib was 58% (range 4–100%). In 89 patients (84.8%), AEs required a dose reduction or interruption of lenvatinib. Receiver operating characteristic curve analysis was performed to determine the optimal 8w-RDI cutoff for predicting objective response, yielding a threshold of 48% (area under the curve = 0.591, 95% CI 0.48–0.70, Supplementary Figure S5). Based on this analysis, a clinically convenient cutoff value of 50% was adopted for further evaluation (sensitivity = 0.88; specificity = 0.40).

**Fig. 3:**
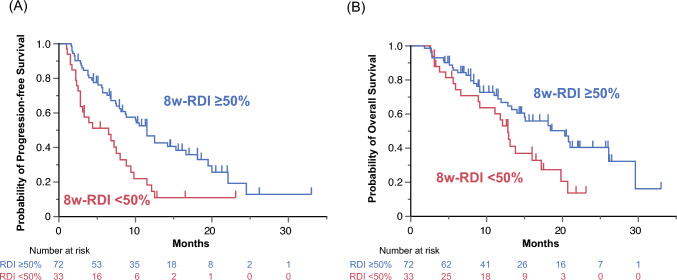
Survival outcomes based on the relative dose intensity of lenvatinib during the initial 8 weeks of treatment. Kaplan–Meier curves for **A** progression-free survival and **B** overall survival are shown. *8w-RDI* the relative dose intensity of lenvatinib during the initial 8 weeks of treatment

Patients with an 8w-RDI of lenvatinib ≥ 50% demonstrated significantly higher ORR (40.3% versus 12.1%, *P* < 0.01) and DCR (84.7% versus 57.6%, *P* < 0.01) compared with those with an 8w-RDI of lenvatinib < 50%. In addition, patients with an 8w-RDI of lenvatinib ≥ 50% exhibited prolonged PFS (HR 0.45, 95% CI 0.28–0.73, Fig. 3A) and OS (HR 0.53, 95% CI 0.30–0.92, Fig. 3B) compared with those with an 8w-RDI of < 50%.

### Prognostic factors for PFS in LP therapy

In the multivariate Cox proportional hazards model conducted to identify the prognostic factors for LP therapy in patients with advanced or recurrent EC, an ECOG-PS of 0 (adjusted HR [aHR] 0.42, 95% CI 0.23–0.75), a PFI of ≥ 6 months (aHR 0.46, 95% CI 0.28–0.78), a histological type of grade 1–2 endometrioid carcinoma (aHR 0.52, 95% CI 0.30–0.91), and an 8w-RDI of lenvatinib ≥ 50% (aHR 0.53, 95% CI 0.31–0.91) were identified as factors associated with improved PFS (Table 3).

**Table 3:** Prognostic factors of lenvatinib plus pembrolizumab therapy for progression-free survival

Factors	Univariate analysis			Multivariate analysis		
	HR	95% CI	*P* value	Adjusted HR	95% CI	*P* value
Age (< 65/≥ 65)	0.79	0.50–1.26	0.32	0.69	0.42–1.14	0.15
ECOG-PS (0/1)	0.41	0.24–0.71	< 0.01	0.42	0.23–0.75	< 0.01
PFI (≥ 6 months/< 6 months)	0.44	0.27–0.70	< 0.01	0.46	0.28–0.78	< 0.01
Histological type (EM G1-2/non-EM G1-2)	0.47	0.29–0.78	< 0.01	0.52	0.30–0.91	0.02
FIGO stage (I–II/III–IV)	0.74	0.45–1.22	0.23	0.76	0.45–1.29	0.30
MMR-status (dMMR/pMMR or unknown)	0.45	0.11–1.84	0.27	0.55	0.13–2.32	0.41
8w-RDI of lenvatinib (≥ 50%/< 50%)	0.45	0.28–0.73	< 0.01	0.53	0.31–0.91	0.02

*HR* hazard ratio, *CI* confidence interval, *ECOG-PS* Eastern Cooperative Oncology Group performance status, *PFI* platinum-free interval, *EM G1–2* grade 1–2 endometrioid carcinoma, *FIGO* the International Federation of Gynecology and Obstetrics, *MMR* mismatch repair, *dMMR* MMR-deficient, *pMMR* MMR-proficient, *8w-RDI* the relative dose intensity during the initial 8 weeks of treatment

### AEs associated with LP therapy

The frequency and time to onset of AEs associated with LP in the 105 patients enrolled in this study are summarized in Supplementary Table S3. All patients experienced at least one AEs, with hypertension being the most common (72.4%, 76/105 patients) and having the shortest time to onset, with a median of 9 days. In addition to hypertension, the AEs occurring in more than 50% of patients were hypothyroidism (68.6%), fatigue (54.3%), proteinuria (53.3%), and decreased platelet count (51.4%). Although less frequent, serious AEs included fistula formation in seven patients (6.7%), colitis in six patients (5.7%), and adrenal insufficiency in four patients (3.8%).

With regard to the association between AEs and PFS, the occurrence of hand-foot syndrome (HFS), also known as palmar-plantar erythrodysesthesia, was significantly associated with improved PFS (HR 0.31, 95% CI 0.18–0.55) and OS (HR 0.30, 95% CI 0.15–0.59) (Supplementary Figure S6). No other AEs demonstrated a significant association with survival outcomes in the study population.

## Discussion

### Main findings

The key findings of this study are as follows. First, several factors were independently associated with improved PFS during LP therapy for advanced or recurrent EC, including an ECOG-PS of 0, a PFI of ≥ 6 months, a histological subtype of grade 1–2 endometrioid carcinoma, and an 8w-RDI of lenvatinib ≥ 50%. Second, both a PFI of ≥ 6 months and an 8w-RDI of lenvatinib ≥ 50% were associated with significantly improved PFS, OS, and ORR.

### Comparison with existing literature

#### PFI in the treatment of EC

A retrospective study in Japan involving 262 patients with recurrent EC who had received first-line platinum-based chemotherapy and were subsequently treated with second-line platinum-based chemotherapy demonstrated that a PFI of < 12 months was associated with significantly shorter PFS (4.4 months versus 10.3 months, *P* < 0.01) and OS (13.8 months versus 40.9 months, *P* < 0.01) compared with a PFI of ≥ 12 months [7]. These results suggested that PFI may affect the therapeutic efficacy of second-line platinum-based chemotherapy in recurrent EC. However, the influence of PFI on outcomes in patients treated with LP therapy remains unclear. Therefore, the findings of this study, which showed that PFI influenced not only OS and PFS but also DOR and tumor response in patients receiving LP therapy, may have meaningful clinical implications in guiding the selection of therapeutic agents for patients with recurrent EC following chemotherapy.

Among patients with recurrent EC who are challenging to treat, those with a PFI of < 6 months are considered to have a markedly poor prognosis. This study found that patients with a PFI of < 6 months who received LP demonstrated unfavorable outcomes, with a median PFS of 5.7 months (95% CI 3.3–7.9) and a median OS of 11.9 months (95% CI 7.9–16.0). These findings are consistent with those of a multicenter retrospective study in Japan, which reported that patients with a PFI of < 6 months who received second-line platinum-based chemotherapy had a median PFS of 3.2 months (95% CI 2.3–4.3) and a median OS of 11.3 months (95% CI 7.9–17.5) [7]. Additionally, our group previously conducted a phase I/II trial evaluating a combination chemotherapy regimen consisting of gemcitabine, levofolinate, irinotecan, and 5-fluorouracil in patients with recurrent EC and a PFI of < 6 months. In that study, the median PFS was 3 months (95% CI 3–7), and the median OS was 12 months (95% CI 9–17) [14]. Taken together, these findings underscore the limited efficacy of currently available treatment options for patients with recurrent EC and a short PFI, highlighting the urgent need to develop novel therapeutic agents.

#### LP therapy for patients with UCS

This study included 10 patients with UCS who were excluded from the previous phase III Study 309/KEYNOTE-775 trial. The ORR and DCR of LP therapy in these patients were 30% and 70%, respectively. These outcomes are consistent with previously reported retrospective data. A Japanese retrospective study of 5 patients with UCS treated with LP therapy reported an ORR of 40% and a DCR of 60% [15], whereas a retrospective study of 12 patients with UCS conducted in the US reported an ORR of 25% and a DCR of 58.3% [16].

In this study, patients with UCS had a significantly shorter PFS compared with those with grade 1–2 endometrioid carcinoma. However, given the small sample size and limited observation period of this study, further large-scale investigations are warranted to draw definitive conclusions regarding the efficacy of LP therapy in patients with UCS.

#### Effect of lenvatinib dosage on treatment efficacy

Several studies have indicated a correlation between the dosage of tyrosine kinase inhibitors (TKIs), such as lenvatinib and sunitinib, and their anti-tumor efficacy [17, 18]. In a multicenter retrospective study conducted in Japan involving 50 patients with hepatocellular carcinoma, those with an 8w-RDI of lenvatinib ≥ 75% exhibited significantly prolonged PFS compared with those with an 8w-RDI < 75% (7.4 months [95% CI 5.9–9.8] versus 3.3 months [95% CI 1.4–5.8], *P* < 0.01) [19]. Similarly, a retrospective study of 49 patients with thyroid cancer demonstrated that an 8w-RDI of lenvatinib ≥ 60% was associated with improved OS (HR 0.31, 95% CI 0.11–0.91) and PFS (HR 0.29, 95% CI 0.11–0.72) compared with an 8w-RDI < 60% [20]. Although this association had not been previously established in EC, the present study demonstrated that patients with an 8w-RDI of lenvatinib ≥ 50% experienced significantly improved PFS and OS compared with those with an 8w-RDI < 50%. These findings suggest that the dose intensity of lenvatinib may influence clinical outcomes in patients with EC.

During chemotherapy with lenvatinib, AEs often require temporary treatment interruptions or dose reductions. In the present study, all patients experienced at least one AEs, and 84.8% (89 of 105 patients) required either a reduction or an interruption of lenvatinib due to AEs. In hepatocellular carcinoma, a weekends-off dosing strategy—administering lenvatinib for 5 days followed by a 2-day rest period—has been reported to improve tolerability to AEs and contribute to prolonged treatment duration and OS [21]. Accordingly, further studies are needed to determine optimal lenvatinib dosing strategies that maintain the RDI while effectively managing AEs in patients with EC.

#### Association between AEs and oncological outcomes

Lenvatinib is a TKI that exhibits antitumor activity by inhibiting multiple receptors, including vascular endothelial growth factor receptor (VEGFR), fibroblast growth factor receptor, and platelet-derived growth factor receptor (PDGFR) [22]. The inhibition of these signaling pathways contributes to a variety of AEs, including hypertension, hypothyroidism, and HFS. In the present study, patients with EC who experienced HFS during LP therapy exhibited significantly prolonged PFS and OS. Although the precise mechanism underlying TKI-induced HFS remains unclear, it is believed to involve the combined inhibition of VEGFR and PDGFR. The blockade of these pathways interferes with vascular repair processes in high-pressure areas, such as the palms and soles, resulting in skin reactions [23, 24].

Several studies have reported a correlation between lenvatinib-related AEs and improved patient prognosis. An exploratory analysis of a phase III trial in patients with thyroid cancer treated with lenvatinib demonstrated that treatment-emergent hypertension was significantly associated with prolonged OS (HR 0.43, 95% CI 0.27–0.69) [25]. Similarly, in a prospective study conducted in Japan, patients with advanced hepatocellular carcinoma who developed grade 2–3 hypothyroidism as a lenvatinib-related AE exhibited improved OS compared with those with grade 1 or lower hypothyroidism (HR 0.21, 95% CI 0.05–0.94) [26]. These findings suggest that patients who experience TKI-induced AEs may have increased sensitivity to TKIs, potentially resulting in greater therapeutic benefits. However, in the present study, no significant association was observed between lenvatinib-related AEs other than HFS and survival outcomes. Further studies are warranted to elucidate the relationship between treatment-related AEs and oncologic prognosis in patients with EC receiving TKI-based therapy.

### Strengths and limitations

Reports on the outcomes of LP therapy for patients with EC in real-world settings remain limited. This study identified novel prognostic factors for LP therapy, including a PFI of ≥ 6 months, grade 1–2 endometrioid carcinoma, and an 8w-RDI of lenvatinib of ≥ 50%. These findings may influence the selection of therapeutic agents for the management of recurrent EC following chemotherapy.

However, this study also has several limitations. First, as an observational study that did not require intervention, an unmeasurable bias may exist concerning the indications for LP therapy and decisions regarding dose reduction or interruption. Second, imaging evaluation was not standardized and was performed at the discretion of the attending physician at each participating institution; this variability may have affected the accuracy of measurements for DOR and time to response, and should be considered when interpreting these outcomes. Third, this study was not a comparative analysis of treatment efficacy between LP and other regimens, such as paclitaxel plus carboplatin (TC); therefore, we could not directly prioritize regimen selection for advanced or recurrent EC. Larger prospective studies are required to resolve this issue.

Fourth, in contrast to clinical trials, this study included 41 patients (39.0%) whose MMR status was not assessed. The lack of a significant association between MMR status and prognosis observed in this study may be attributed to the limited number of patients with confirmed MMR deficiencies. Nevertheless, the inclusion of patients who underwent LP therapy without MMR testing reflects real-world clinical practice in Japan, where treatment options for second-line chemotherapy in advanced or recurrent EC are more limited than in other countries. Fifth, the number of events observed in the OS analysis was lower than that in the PFS analysis, owing to the limited sample size and relatively shorter observation period. Consequently, a multivariate analysis for prognostic factors associated with OS was not performed. To address this limitation, extended follow-up and additional long-term data collection are planned to enable further analyses of OS outcomes.

Sixth, although this study investigated the site of recurrence, the number and extent of metastatic lesions were not evaluated. Therefore, it was difficult to comprehensively analyze the relationship between the recurrence site and prognosis in patients treated with LP therapy. Further research is needed to clarify the effect of recurrence burden and distribution on treatment outcomes. Finally, the BOR was assessed using either RECIST version 1.1 or iRECIST, based on the discretion of the attending physician. Given that pseudoprogression has been reported in patients with lung cancer and melanoma receiving ICIs [27, 28], iRECIST was permitted for patients receiving pembrolizumab in this study. However, the use of two different assessment criteria may have introduced variability and affected the accuracy of the BOR evaluations. Future studies are recommended to evaluate BOR using both criteria to compare their differences.

### Implications for practice and future research

This multicenter observational study in Japan identified potential prognostic factors of LP therapy in patients with advanced or recurrent EC. The observed association between the PFI and survival outcomes, including tumor response, may prove useful in predicting the efficacy of LP therapy in patients experiencing recurrence following chemotherapy. However, this study and previous phase III trials investigating the efficacy of LP therapy, including Study 309/KEYNOTE-775 and LEAP-001 trials, excluded patients with a history of ICI use [8, 29]. Recently, several phase III trials reported that TC combined with ICIs, such as pembrolizumab, durvalumab, and dostarlimab, was more effective compared with conventional TC as first-line chemotherapy for advanced or recurrent EC [30–32]. As these treatments become more widely used, the proportion of relapsed patients who have previously received ICIs is expected to increase in the future. Therefore, further research is needed to evaluate the efficacy of LP therapy in patients with EC who experienced disease recurrence after ICI use.

## Supplementary Information

Below is the link to the electronic supplementary material.

Supplementary file1 (PDF 962 KB)

## Data Availability

The dataset used and/or analyzed during the current study are available from the corresponding author on reasonable request.
