# Supplementary material for: Prognostic factors of lenvatinib plus pembrolizumab therapy for advanced or recurrent endometrial cancer: analysis of a multicenter cohort study in Japan
Source: Int J Clin Oncol. 2025 Sep 6;30(11):2342–51. doi: 10.1007/s10147-025-02842-x (PMC12568866; doi:10.1007/s10147-025-02842-x)

Supplementary Table S1. Chemotherapy regimen prior to lenvatinib plus pembrolizumab therapy

	N (%)
Carboplatin/ Paclitaxel	81 (77.1)
Carboplatin/ Epirubicin/ Paclitaxel	26 (24.8)
Carboplatin/ Docetaxel	15 (14.3)
Cisplatin/ Doxorubicin	12 (11.4)
Gemcitabine/ Levofolinate/ Irinotecan/ 5-Fluorouracil	3 (2.9)
Carboplatin/ Doxorubicin/ Paclitaxel	2 (1.9)
Cisplatin/ Docetaxel	2 (1.9)
Cisplatin/ Paclitaxel	1 (1.0)
Doxorubicin	1 (1.0)
Carboplatin	1 (1.0)

Supplementary Table S2. Subsequent therapy after lenvatinib plus pembrolizumab therapy

	N
Chemotherapy	
Cisplatin/ Doxorubicin	6
Carboplatin/ Paclitaxel	5
Carboplatin/ Epirubicin/ Paclitaxel	2
Carboplatin/ Docetaxel	1
Doxorubicin	4
Pembrolizumab	1
Other	1
Radiotherapy	3
Hormonal therapy	
Medroxyprogesterone acetate	2

Supplementary Table S3. Adverse events of lenvatinib plus pembrolizumab therapy

	Any grade<br>n (%)	Grade 3≤<br>n (%)	Time to onset of AEs<br>day, median (range)
Hypertension	76 (72.4)	40 (38.1)	9 (1-365)
Hypothyroidism	72 (68.6)	1 (1.0)	44.5 (1-410)
Fatigue	57 (54.3)	13 (12.4)	37 (1-367)
Proteinuria	56 (53.3)	13 (12.4)	43 (9-408)
Platelet count decreased	54 (51.4)	10 (9.5)	22.5 (6-504)
Anorexia	43 (41.0)	7 (6.7)	46 (3-413)
Aspartate aminotransferase increased	39 (37.1)	4 (3.8)	31 (3-312)
Alanine aminotransferase increased	37 (35.2)	4 (3.8)	26 (1-503)
Hand-foot syndrome	35 (33.3)	9 (8.6)	81 (7-374)
Anemia	34 (32.4)	6 (5.7)	65.5 (1-560)
Diarrhea	28 (26.7)	10 (9.5)	110 (1-539)
Nausea	21 (20.0)	2 (1.9)	27 (1-239)
Hoarseness	21 (20.0)	1 (1.0)	29 (3-370)
Mucositis oral	20 (19.0)	3 (2.9)	68.5 (8-502)
Neutrophil count decreased	19 (18.1)	2 (1.9)	28 (4-385)
Vomiting	17 (16.2)	3 (2.9)	38 (1-265)
Weight loss	17 (16.2)	1 (1.0)	49 (14-734)
Arthralgia	16 (15.2)	2 (1.9)	53 (12-286)
Hyperthyroidism	10 (9.5)	0 (0)	62.5 (14-96)

Abbreviation: AE, adverse event.

Supplementary Figure S1. Best percentage change from baseline in all patients with measurable target lesions

Waterfall plots demonstrate the best percentage change from baseline for measurable target lesions, based on assessments per RECIST version 1.1 or iRECIST.

Abbreviations: CR, complete response; PR, partial response; SD, stable disease; PD, progressive disease; RECIST, Response Evaluation Criteria in Solid Tumors**.**

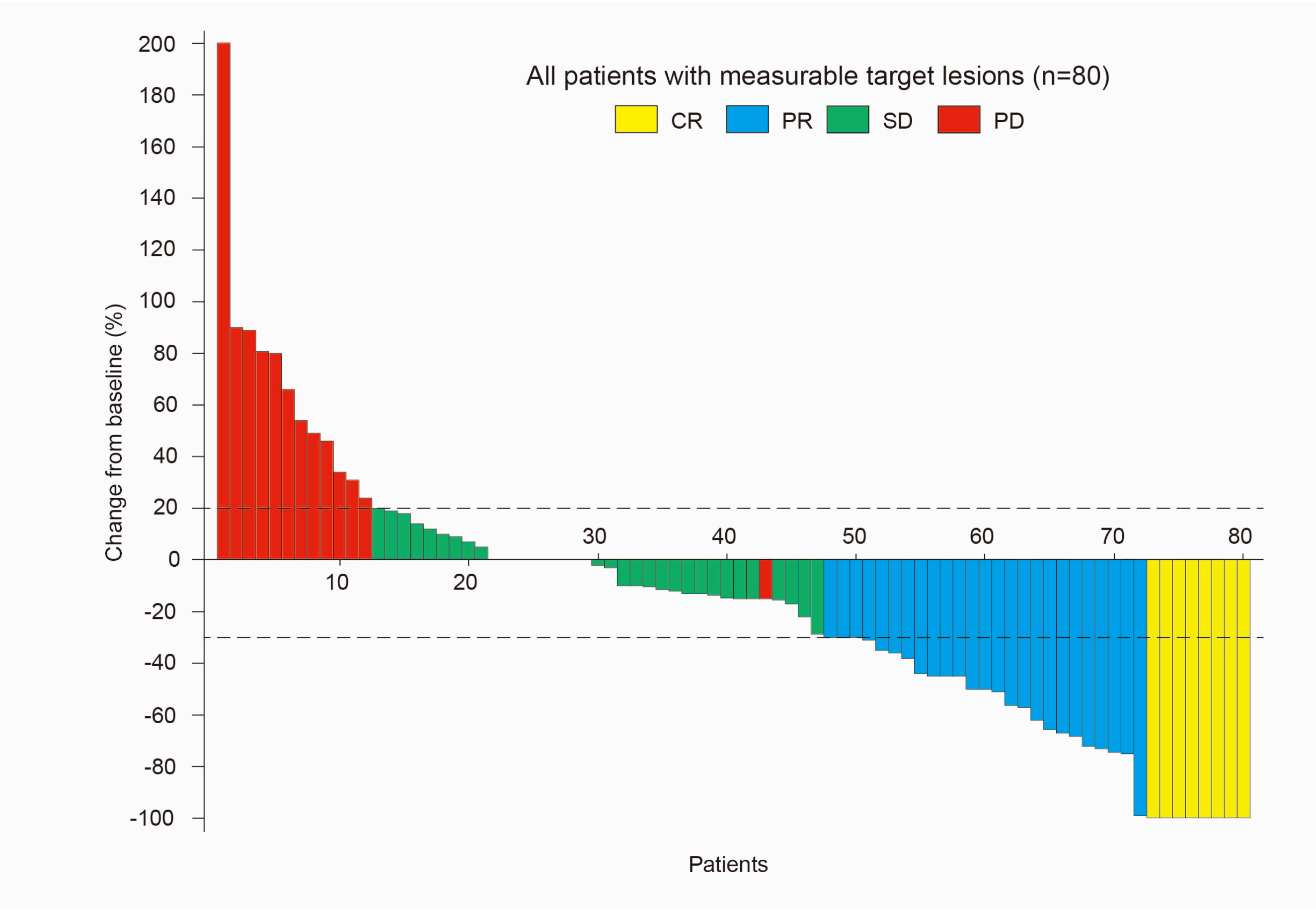

Supplementary Figure S2. Progression-free survival based on the histological types of tumors

Kaplan-Meier curves for progression-free survival in patients with EM G1-2 and (A) non-EM G1-2, (B) EM G3, (C) UCS, or (D) serous carcinoma are shown. Abbreviations: EM G1-2, grade 1-2 endometrioid carcinoma; EM G3, grade 3 endometrioid carcinoma; UCS, uterine carcinosarcoma.

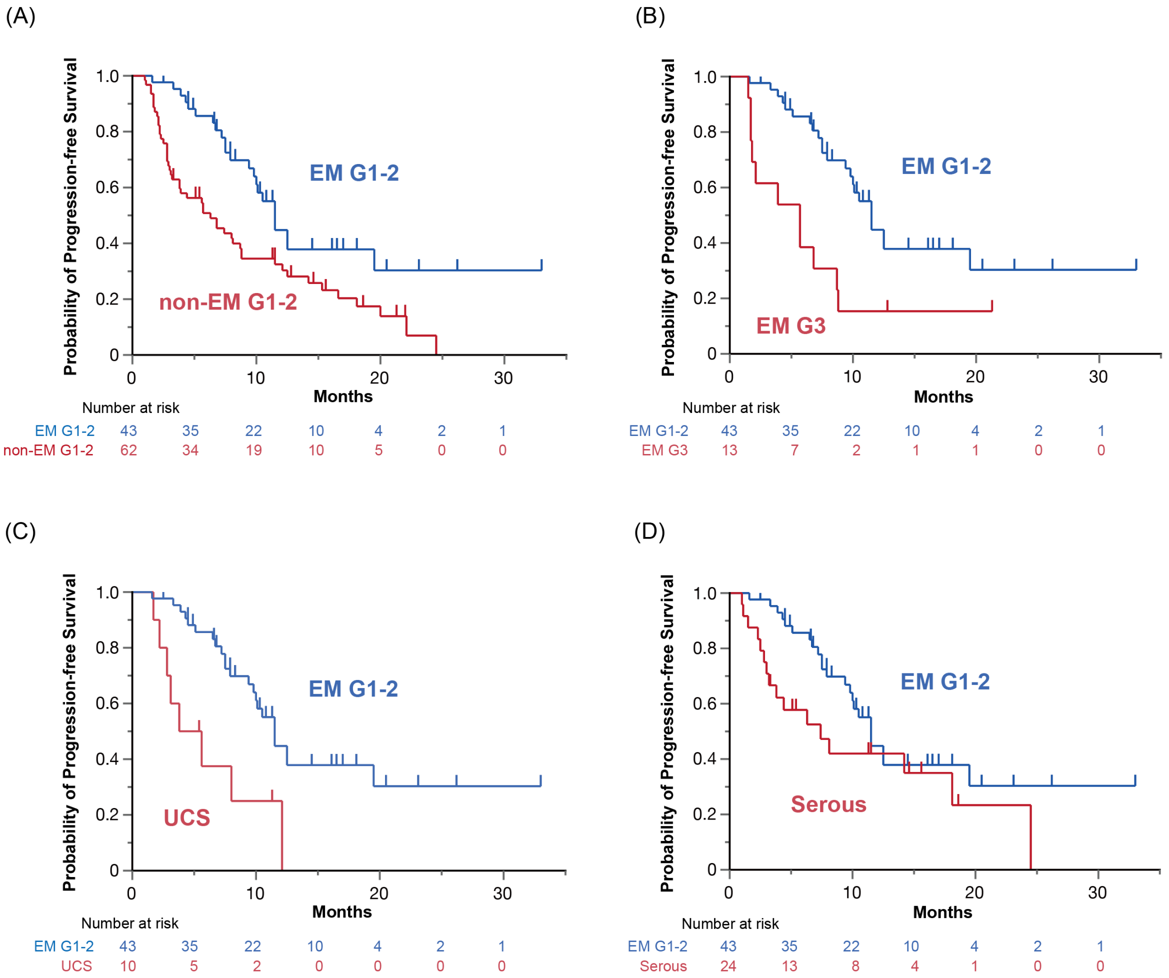

Supplementary Figure S3. Progression-free survival based on the mismatch repair status

Abbreviations: dMMR, mismatch repair-deficient; pMMR, mismatch repair-proficient.

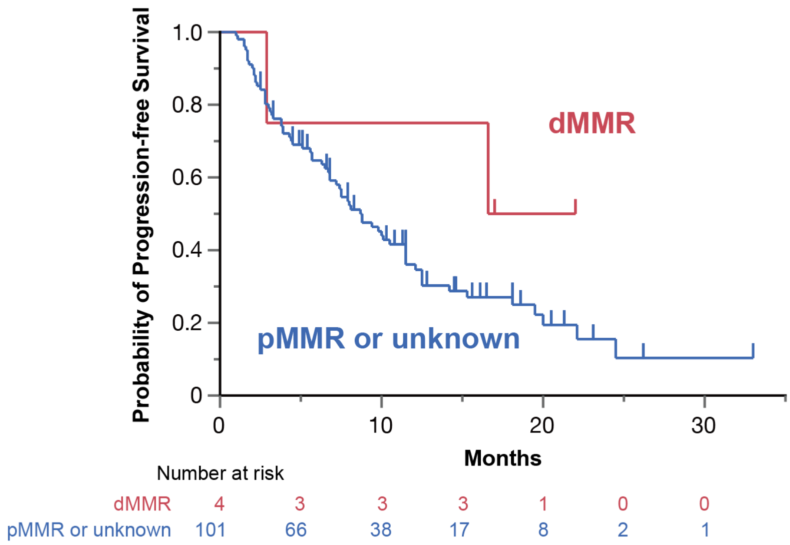

Supplementary Figure S4. Duration of response based on the platinum-free interval in patients who achieved a complete or partial response

Kaplan-Meier curve for duration of response is shown. Abbreviations: PFI, platinum-free interval.

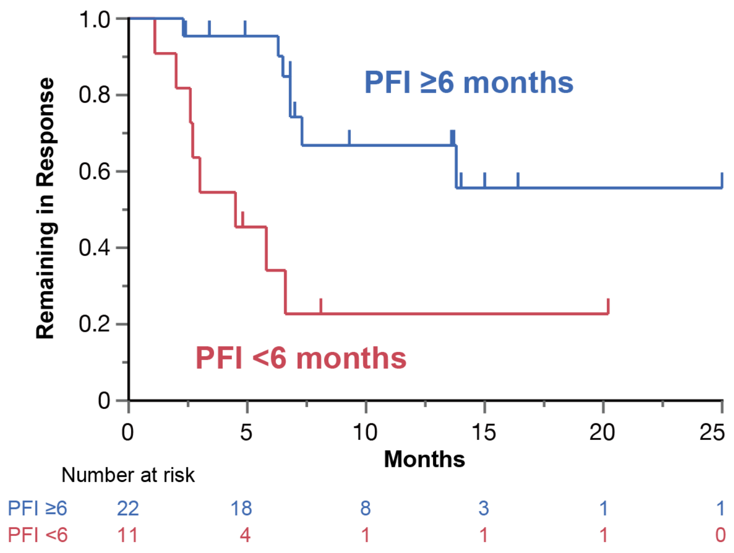

Supplementary Figure S5. Receiver operating characteristic curve for the relative dose intensity of lenvatinib during the initial 8 weeks concerning objective response

Cutoff value of the relative dose intensity of lenvatinib was determined to be 48% by analysis using the receiver operating characteristic curve (area under the curve = 0.591, 95% confidence interval 0.48-0.70).

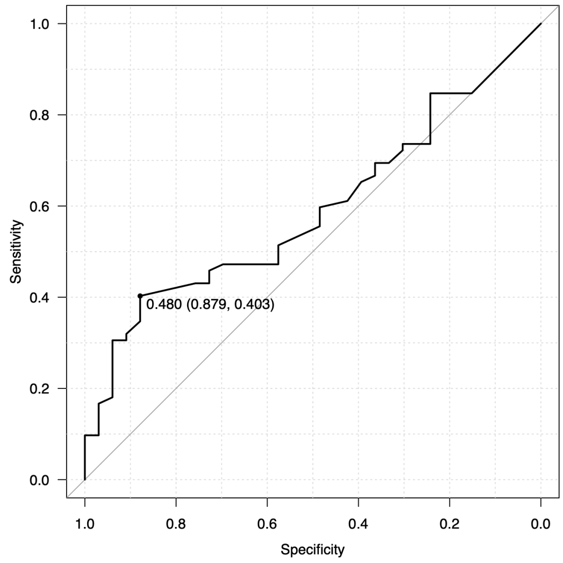

Supplementary Figure S6. Survival outcomes based on the presence or absence of hand-foot syndrome in patients who received lenvatinib plus pembrolizumab

Kaplan-Meier curves for (A) progression-free survival and (B) overall survival are shown. Abbreviations: HFS, hand-foot syndrome.

(A)

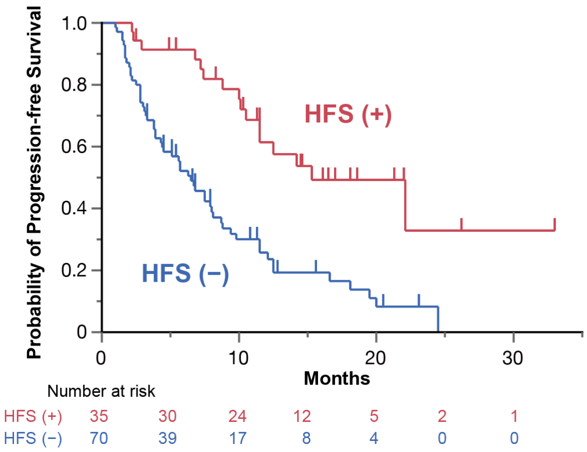

(B)

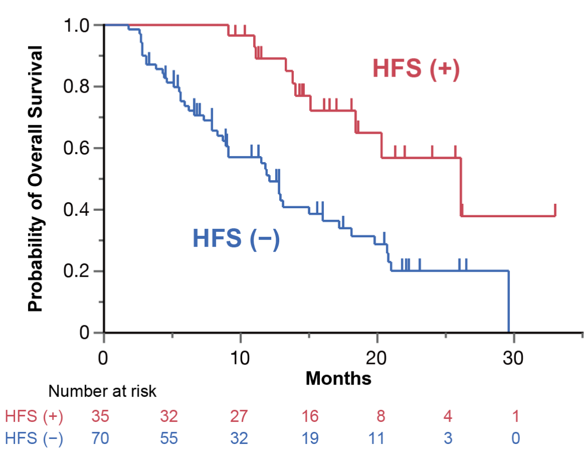

Supplement: Supplementary file 1 — Supplementary file1 (PDF 962 KB) [file 10147_2025_2842_MOESM1_ESM.pdf]
